# Further confirmation of the association of *SLC12A2* with non-syndromic autosomal-dominant hearing impairment

**DOI:** 10.1038/s10038-021-00954-6

**Published:** 2021-07-05

**Authors:** Samuel M. Adadey, Isabelle Schrauwen, Elvis Twumasi Aboagye, Thashi Bharadwaj, Kevin K. Esoh, Sulman Basit, Anushree Acharya, Liz M. Nouel-Saied, Khurram Liaqat, Edmond Wonkam-Tingang, Shaheen Mowla, Gordon A. Awandare, Wasim Ahmad, Suzanne M. Leal, Ambroise Wonkam

**Affiliations:** 1 West African Centre for Cell Biology of Infectious Pathogens (WACCBIP), University of Ghana, Accra, Ghana; 2 Division of Human Genetics, Faculty of Health Sciences, University of Cape Town, Cape Town, South Africa; 3 Center for Statistical Genetics, Gertrude H. Sergievsky Center, and the Department of Neurology, Columbia University Medical Centre, New York, NY USA; 4 Center for Genetics and Inherited Diseases, Taibah University Al Madinah Al Munawarah, Al Munawarah, Saudi Arabia; 5 Faculty of Biological Sciences, Department of Biotechnology, Quaid-i-Azam University, Islamabad, Pakistan; 6 Division of Haematology, Faculty of Health Sciences, Department of Pathology, University of Cape Town, Cape Town, South Africa; 7 Faculty of Biological Sciences, Department of Biochemistry, Quaid-i-Azam University, Islamabad, Pakistan; 8 Taub Institute for Alzheimer’s Disease and the Aging Brain, Columbia University Medical Centre, New York, NY USA

**Keywords:** Disease genetics, Neurological disorders

## Abstract

Congenital hearing impairment (HI) is genetically heterogeneous making its genetic diagnosis challenging. Investigation of novel HI genes and variants will enhance our understanding of the molecular mechanisms and to aid genetic diagnosis. We performed exome sequencing and analysis using DNA samples from affected members of two large families from Ghana and Pakistan, segregating autosomal-dominant (AD) non-syndromic HI (NSHI). Using in silico approaches, we modeled and evaluated the effect of the likely pathogenic variants on protein structure and function. We identified two likely pathogenic variants in *SLC12A2*, c.2935G>A:p.(E979K) and c.2939A>T:p.(E980V), which segregate with NSHI in a Ghanaian and Pakistani family, respectively. *SLC12A2* encodes an ion transporter crucial in the homeostasis of the inner ear endolymph and has recently been reported to be implicated in syndromic and non-syndromic HI. Both variants were mapped to alternatively spliced exon 21 of the *SLC12A2* gene. Exon 21 encodes for 17 residues in the cytoplasmatic tail of SLC12A2, is highly conserved between species, and preferentially expressed in cochlear tissues. A review of previous studies and our current data showed that out of ten families with either AD non-syndromic or syndromic HI, eight (80%) had variants within the 17 amino acid residue region of exon 21 (48 bp), suggesting that this alternate domain is critical to the transporter activity in the inner ear. The genotypic spectrum of *SLC12A2* was expanded and the involvement of *SLC12A2* in ADNSHI was confirmed. These results also demonstrate the role that *SLC12A2* plays in ADNSHI in diverse populations including sub-Saharan Africans.

## Introduction

Hearing impairment (HI) is the most common sensory human disorder [1]. Although the characterization of HI is complex, it may be described as conductive, sensorineural, or mixed; syndromic or non-syndromic (NS); prelingual or post-lingual, progressive, or nonprogressive [2]. The etiology of HI stems from environmental (birth complications, some infectious diseases, chronic ear infections, ototoxic medications, accidents, exposure to excessive noise, and aging) and genetic/inheritable factors [1]. HI genetics is highly heterogeneous [2]; to date, >120 genes have been implicated in NSHI [3]. Pathogenic variants in the gap junction protein beta 2 (*GJB2)*, encoding for connexin 26, remain the most common genetic cause of NSHI [4].

The advancements in next-generation sequencing techniques have accelerated the discovery of HI-associated gene variants [5–7]. In this report, we used exome sequencing (ES) to identify pathogenic variants in *SLC12A2* in two families, one Ghanaian and the other Pakistani, both segregating autosomal-dominant (AD) NSHI. The SLC12A are transmembrane proteins that mediate electro-neutral transport of ions, thus influx and efflux of Na^+^, K^+^, and Cl^−^ ions [8]. They regulate physiological function, including ion transport, modulate inhibitory synaptic transmission, and maintain and regulate cell volume [9].

## Materials and methods

### Participants’ enrollment

**Fig. 1:**
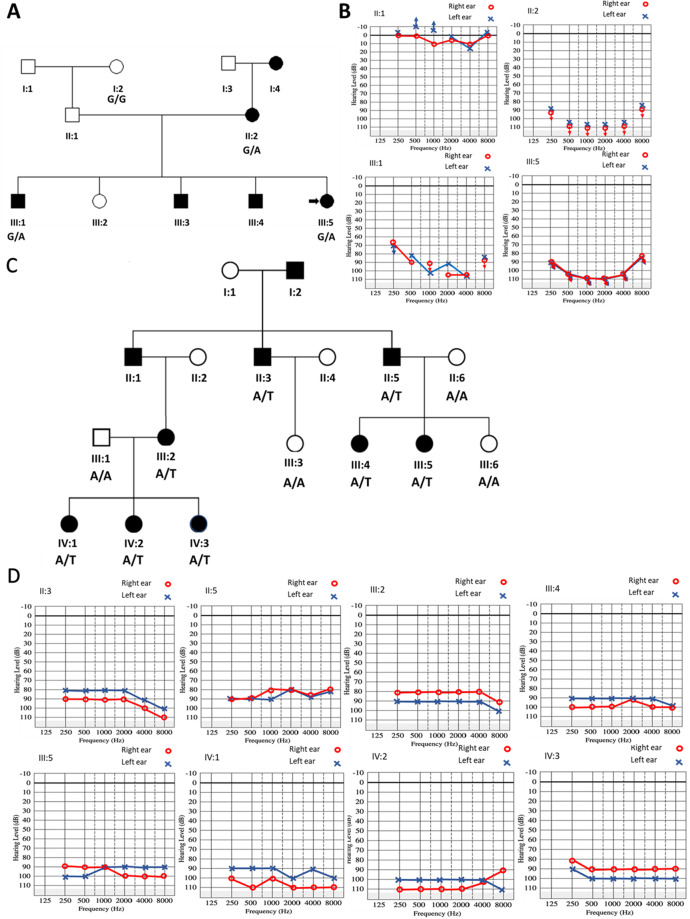
Pedigree and pure-tone audiometry measurements of affected families. **A** Pedigree and **B** audiograms of some individuals in the Ghanaian family (GH-F4), which segregates the *SLC12A2*: c.2935G>A: p.(E979K) variant. The ages of affected individuals: II:2, III:1, and III:5 in Fam GH-F4 at the time of sample collection were 29, 10, and 3 years, respectively. **C** Pedigree and **D** audiograms of some individuals in the Pakistan family (PK-4543) with *SLC12A2*: c.2939A>T: p.(E980V) variant. The ages of affected individuals in family PK-4543: II:3, II:5, III:2, III:4, III:5, IV:1, IV:2, and IV:3 at the time of sample collection were 56, 60, 38, 35, 37, 10, 11, and 13 years, respectively. The black shaded square and circles were used to denote hearing-impaired males and females, respectively. The unshaded squares and circles correspond to hearing males and females

Two families that reported congenital HI were recruited from Ghana (GH-F4) and Pakistan (PK-4543; Fig. 1A, C). The Ghanaian family (GH-F4) participated in a previous study that screened hearing-impaired families from Ghana for *GJB2* mutations [10]. A structured questionnaire and a review of the medical records of the participant were employed to rule out environmental or syndromic HI and pure-tone audiometry was performed.

Pakistani family (PK-4543) is from the Mardan district in the Khyber Pakhtunkhwa province of Pakistan. The patient evaluation included a clinical history, physical, audiological, and vestibular examination, including pure-tone audiometry and a Romberg test. Environmental causes of HI, such as maternal or perinatal infections, administration of ototoxic medications, or trauma were excluded.

### Sample collection and initial screening

Peripheral blood samples were collected for genomic DNA (gDNA) isolation for 4 family members (3 affected and 1 unaffected) from GH-F4 (I:2, II:2, III:1, and III:5) and for 12 family members (8 affected and 4 unaffected) from Pakistani pedigree PK-4543 (II:3, II:5, II:6, III:1–6, and IV:1–3). gDNA isolation was performed using the QIAmp DNA Mixi Prep Kit and a phenol–chloroform protocol [11] for GH-F4 and PK-4543, respectively. Prior to exome sequencing, the entire coding region of *GJB2* was screened using Sanger sequencing in both families. Additional common HI-associated variants in *CIB2*, *HGF*, and *SLC26A4* in the Pakistani population were also screened via Sanger sequencing in family PK-4543, as previously described [7].

### Exome sequencing

Exome sequencing was performed on selected samples from each family (GH-F4: II:2, III:1, and III:5 and PK-4543: III:5 and IV:2). The gDNA samples were fragmented and a library was prepared using the Illumina Nextera Rapid Capture Exome kit for family GH-F4 (37-Mb target region, Illumina, San Diego, CA, USA) and the SureSelect Human All Exon V6 kit for family PK-4543 (60.5-Mb target region, Agilent Technologies, Santa Clara, CA, USA). Paired-end sequencing was performed on a HiSeq2500/4000 instrument (Illumina Inc, San Diego, CA, USA).

Reads were aligned to the human reference genome (GRCh37/Hg19) using Burrows–Wheeler Aligner-MEM for family PK-4543 [12] and the Dynamic Read Analysis for GENomics (DRAGEN 05.021.408.3.4.12) software for GH-F4 (Illumina Inc, San Diego, CA, USA). Duplicate removal, insertions/deletion (Indel)-realignment, and base quality score recalibration were performed with Picard-tools and the Genome Analysis Toolkit (GATK) [13] (for PK-4543) and the DRAGEN software (for GH-F4). Single-nucleotide variants and InDels were jointly called by the GATK HaplotypeCaller [13]. The sex of each individual with exome data was verified using plinkv1.9 [14]. Familial relationships for members with exome data were verified via Identity-by-Descent sharing (plinkv1.9) and the Kinship-based INference for Gwas algorithm [15].

An in-house-developed pipeline based on the ANNOVAR [19] tool was used for annotation and filtering. In brief, the analysis was prioritized for AD mode of inheritance, and variants filtering was conducted using a minor allele frequency <0.0005 in each population included in the genome aggregation database (gnomAD) [16]. Candidate genes that are associated with HI were prioritized, based on their known association with HI on the hereditary hearing loss homepage, Online Mendelian Inheritance in Man (OMIM), human phenotype ontology, and ClinVar databases [17].

### Confirmation of identified candidate variants from WES analysis

Sanger sequencing was used to confirm and verify segregation of the identified *SLC12A2* [NM_001046.2:c.2935G>A:p.(E979K) and c.2939A>T: p.(E980V)] variants in families GH-F4 and PK-4543, respectively, using DNA samples from all available family members. Sanger sequencing of the PCR amplicons containing the genomic region of the variants was performed using the BigDye^TM^ Terminator v3.1 Cycle Sequencing Kit. These products were next analyzed on an ABI 3130XL Genetic Analyzer^®^ (Applied Biosystems, Foster City, CA, USA). We used FinchTV v1.4.0, UGENE v34.0 [18], and CodonCode Aligner to analyze the Sanger sequence data. The variants were classified according to the American College of Medical Genetics and Genomics (ACMG) guidelines for hearing loss [19, 20].

### Secondary structure prediction and protein modeling

The wild-type SLC12A2 protein sequence was retrieved from the NCBI database. The PSIPRED v4.0 program (web interphase) [21] was used to predict the effect of the variant on the secondary structural features of SLC12A2. Multiple template-based modeling of both wild-type and mutant structures using the top four PDB hits (6NPJ, 6NPL, 6PZT, and 6UKN) revealed that these PDB structures are not suitable templates for the full-length SLC12A2 protein. More so, the recently solved structure of the SLC12A2 protein is a homodimer of the transmembrane region, which does not contain the variant position of interest. Therefore, a de novo modeling strategy was employed using trRosetta [22]. In brief, 280 N-terminal residues (the N-terminal domain of SCL12A2 isoforms is highly diverse and thought to play no role in ion transportation) of the wild-type and mutant proteins were truncated to yield 932 residues required by trRosetta (≤1000aa).

## Results

### Clinical description of families

Two families were investigated, one from Ghana (GH-F4) and the other from Pakistan (PK-4543) (Fig. 1). The Ghanaian family had a familial history of congenital HI, following a likely AD inheritance, from the maternal line (Fig. 1A). Pure-tone audiometry showed a U-shaped bilateral severe-to-profound HI in affected family members (Fig. 1B). Similarly, the mode of inheritance in the Pakistani family was compatible with an AD mode of inheritance (Fig. 1C), with affected individuals displaying bilateral severe-to-profound HI (Fig. 1D), which was congenital and nonprogressive. Affected individuals in both Ghanaian and Pakistanis families did not have any additional phenotypic features.

### Exome sequencing

**Fig. 2:**
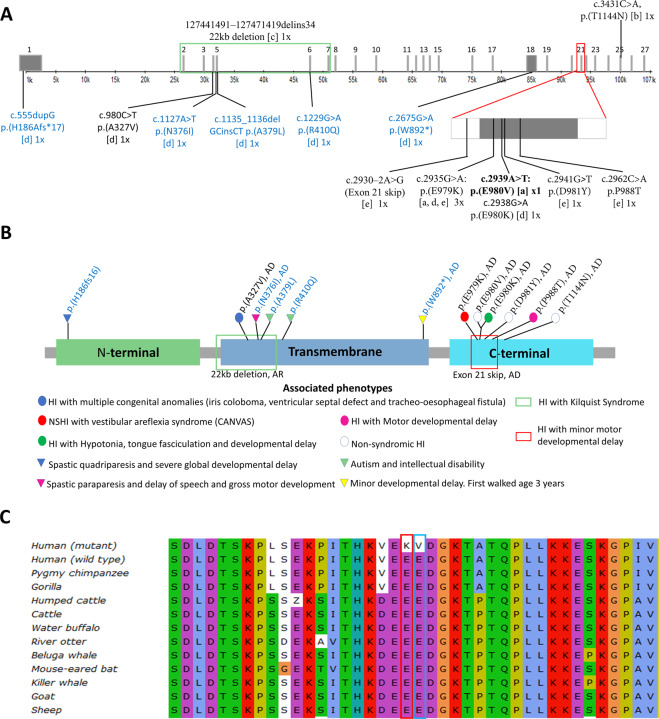
Schematic presentation of SLC12A2 gene and protein alignment. **A** Schematic diagram of HI-associated mutations within the *SLC12A2* gene. The variants comprise of those found in our current study[a] and previous studies in cases that present with both syndromic, and non-syndromic hearing loss: Morgan et al. [29][b], Macnamara et al. [27][c], McNeill et al. [25][d], Mutai et al. [24][e]. The number of independent occurrences (1x or 3x) was written beside each variant. The variants that were not associated with HI were written in blue ink. The exons are denoted with a gray bar with the respective exon number on top of each gray bar. **B** Variants in SLC12A2 protein associated with HI. Circles were used to denote variants associated with HI while triangles were used to represent variants that were not associated with HI. The predominant mode of inheritance was autosomal-dominant (AD), the deletion was autosomal recessive (AR). The mode of inheritance of p.(H196fs16), p.A379L), and p.(R410Q) was not stated by the authors. **C** SLC12A2 protein sequence alignment. The amino acid position of SLC12A2: p.(E979K) and p.(E980V) are highlighted with red and blue rectangles, respectively

The average read depth was 80X and 65X for the Ghanaian and Pakistani samples, respectively. Filtering analysis of the families identified heterozygous missense variants in exon 21 of *SLC12A2* (OMIM:600840): NM_001046.2: c.2935G>A: p.(E979K) for family GH-F4 and c.2939A>T: p.(E980V) for family PK-4543. Each variant was verified with Sanger sequencing (Fig. 2) and segregated with the HI phenotype (Fig. S1A, B). Both variants are absent from gnomAD [16] and TopMed [23] databases. The variants were predicted to be deleterious by six bioinformatic tools and were classified as likely pathogenic according to ACMG classification for HI (Table S1).

The *SLC12A2*-c.2935G>A: p.(E979K) variant has previously been reported as a de novo variant in a Japanese patient with HI [24] and displayed AD inheritance in a family with HI and vestibular areflexia of unknown ancestry [25]. Sanger sequencing confirmed that the *SLC12A2*-c.2935G>A: p.(E979K) variant was absent in 153 unrelated Ghana cases from simplex families with NSHI, and 46 unrelated ethnolinguistically matched controls, sampled within the Ghana population. The *SLC12A2*-c.2939A>T p.(E980V) variant has not been reported previously, however, a de novo SLC12A2-c.2938G>A p.(E980K) variant where the amino acid change from glutamic acid was to lysine instead of valine was found previously in a boy with bilateral HI, hypotonia, and developmental delay who also had an XYY karyotype [25] (Fig. 2B).

**Fig. 3:**
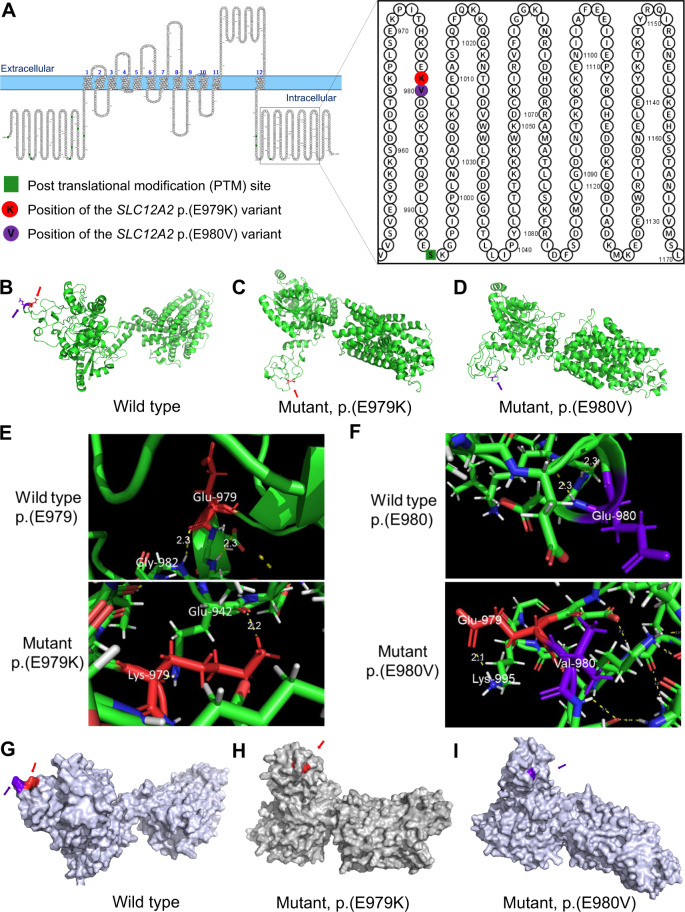
SLC12A2 protein modeling. **A** Primary structure of SLC12A2 protein showing the site of the p.(E979K) and p.(E980V) variants. The ribbon protein model of the **B** wild type, **C** mutant p.(E979K), and **D** mutant p.(E980V) proteins. **E** The variant position showing wild-type residue (E979) and mutant (K979) hydrogen bonds. **F** The variant position showing wild-type residue (E980) and mutant (V980) hydrogen bonds. The surface protein model of the **G** wild type, **H** mutant p.(E979K), and **I** mutant p.(E980V) proteins. The site of the p.(E979K) variant is highlighted in red (pointed to by the red arrows) and the site of the p.(E980V) variant is highlighted in violet (pointed to by the violet arrows)

Both the p.(E979K) and p.(E980V) variants were classified as likely pathogenic (Table S1) and lie adjacent in an intracellular area of the protein, which is highly conserved amongst species (Fig. 3A). Interestingly, most previously reported variants associated with HI also lie in this exact area, within exon 21 (Fig. 2A) [24, 25], suggesting that this area is critical to SLC12A2 transporter activity.

### Evolutionary conservation of amino acid at position 979 and 980 of SLC12A2 protein

To further evaluate the evolutionary importance of the amino acid at positions 979 and 980 of the protein, a multiple sequence alignment was conducted. It was observed from the sequence alignment that both positions were conserved across the species studied. The conservation of the glutamic acid at these positions suggests their importance to the protein structure and function (Fig. 2C).

### Secondary structure prediction and protein modeling

SLC12A2 is a 1212 amino acid protein made of 12 intermembrane helices and cytoplasmic N- and C-terminal domains. The SLC12A2: p.(E979K) and p.(E980V) variants are located on the C-terminal domain (Fig. 3A). The wild-type glutamate (E) residue falls within a string of three successive E residues (^978^EEE^980^), which, due to repulsion of their electron clouds, likely prevents the formation of any secondary structural feature in the region. The variants in these positions therefore disrupt this pattern, introducing a positively charged (lysine) residue in the p.(E979K) mutation and a hydrophobic (valine) residue in the p.(E980V) mutation, which may explain the formation of short helices in both mutants (Fig. S2). Further analysis of the variants on Expasy-ProtParam indicated that the variants increased the pI [wild type = 5.98; p.(E979K) = 6.08; p.(E980V) = 6.03] and reduced the net charge [wild type = −15; p.(E979K) = −13; p.(E980V) = −14] of the protein. Indeed, the variants were predicted to distort secondary structural features along the entire length of the protein. For instance, formation of the helix structures at 976KVE978 and 977VE978 around the variant positions in the mutant proteins, respectively (Fig. S2, red box on KVE); loss of the helical structure at 987QPLLK991 residues in the p.(E979K) mutant; loss of the strand at 951VVSVE955 residues in the p.(E980V) mutant (Fig. S2, red box on VVSVE); as well as loss of the strand at 283VKF285 residues in both mutants (Fig. S2, red box on VKF). In short, strand and helical propensities in multiple regions of the mutant proteins were affected (Supplementary Fig. S2).

The effects of the secondary structural changes were evident in the wild-type compared to the mutant SLC12A2 3D structures (Fig. 3B–D) modeled with trRosetta and refined using the Galaxy Refine algorithm [26]. In the wild-type structure, the glutamate residue (E979) formed a hydrogen bond with a glycine residue (G982) (Fig. 3E). However, the mutant lysine residue (K979) formed a hydrogen bond with a distant glutamate residue (E942) with a slightly shorter bond length (Fig. 3E). While the V980 mutant formed no side chain bonds, it disrupted the wild-type E979-G982 hydrogen bond and imposed a new E979-K959 hydrogen bond (Fig. 3F). This was consistent with the substantial structural changes seen on the surface view of the protein structures (Fig. 3G–I). These structural differences are likely due to the positive charge introduced by the mutant K residue, or the hydrophobic nature of the mutant V residue. The refined structures were predicted to have ProsA *Z*-scores within expected regions for X-ray experimentally determined structures: Zwt = −9.39; Zp.(E979K) = −11.15; Zp.(E980V) = −11.28 (Supplementary Fig. S3). For all the structures, more than 99% of the residues were in allowed regions in a Ramachandran plot (Supplementary Fig. S3). These values underscore the high quality of the modeled and refined structures.

## Discussion

The *SLC12* family is a nine-member gene family with three established associated human diseases: *SLC12A1* (MIM No. 600839), *SLC12A3* (MIM No. 600968), and *SLC12A6* (MIM No. 604878), which cause Bartter, Gitelman, and Andermann syndromes, respectively [27]. These well-described autosomal recessive (AR) conditions are caused by defects in the movement and regulation of inorganic sodium (*SLC12A1* and *SLC12A3*) and potassium (*SLC12A6*) cations against the movement of chloride anions [8]. SLC12A2, a Na–K–Cl cotransporter (NKCC1), is responsible for chloride transport across cells and supports cell-to-cell communication. It was suggested to be important for the homeostasis of the endolymph by recycling K+ from the perilymph to the stria vascularis in the mammalian cochlea [24, 26, 28]. *SLC12A2* (MIM No. 600840) has recently been reported to be involved in ADNSHI DFNA78 [24, 29], AD Delpire–McNeill syndrome (MIM: 619083), and AR Kilquist syndrome (MIM: 619080). Both syndromes are also associated with HI. In this study, we identified two variants in exon 21 of *SLC12A*2 [c.2935G>A: p.(E979K) and c.2939A>T: p.(E980V)], which segregate with ADNSHI in a Ghanaian and Pakistani family, respectively. The first variant, p.(E979K), is a known pathogenic variant that has been reported previously as de novo variant [24] and segregating in a family with ADNSHI [25]. The second variant, p.(E980V), is novel but a p.(E980K) change was reported in a patient with Delpire–McNeill syndrome, which includes HI [25]. Both amino acids at the variant sites were conserved across several species, which was consistent with the previous report [24]. Both variants are absent from human genome databases including gnomAD and TOPMed, and 398 Ghanaian chromosomes. Our present report further confirms the pathogenic role of *SLC12A2* in HI.

Except for a homozygous deletion reported by Macnamara et al. [27], all the other HI-associated variants (8/9) in the *SLC12A2* gene were inherited in an AD fashion or occurred de novo. It is notable that in the previous and current studies of *SLC12A2*, the majority (*n* = 8/10; 80%) of families and probands with ADNSHI or AD syndromic HI had variants in exon 21 (Fig. 3A). Previous reports identified five variants outside of exon 21 that were not associated with HI [27] (Fig. 2A). Exon 21 encodes for 17 amino acid residues, which created an in-frame alternative domain in the long C-terminal cytoplasmic domain of SLC12A2. This area is conserved between species, but not between SLC12A proteins making it therefore unique to SLC12A2 [24]. A study found that exon 21 displays natural alternative splicing and an isoform lacking exon 21 exist in the developing human brain and other mammalian tissues, with some tissues preferentially expressing the exon 21 skipped short isoform (NP_001243390.1) (such as the mouse cerebellum) [24]. Notably, the longer isoform (NP_001037.1) that includes exon 21 was either the only isoform or the main isoform expressed in cochlear tissues, suggesting that this region confers a tissue-specific function [24]. In vitro evaluation exon 21 [p.(E980K] variant in Xenopus laevis oocytes, showed a reduction in the cotransporter function of SLC12A2 [25]. Suggesting exon 21 may be critical to transporter activity in the inner ear.

Loss of *SLC12A2* (NKCC1) in mice causes sensorineural HI and imbalance problems described as the “shaker/waltzer” phenotype [8]. Additional phenotypes including saliva production, intestinal transit problems, reduced neuron density, and many epithelial-related symptoms have been described in mice [27]. Histological examination of Na–K-Cl cotransporter null mice inner ears showed dysfunctional cochlea with a complete collapse of the cochlear duct [30].

## Conclusion

We identified heterozygous likely pathogenic variants in SLC12A2 (MIM No. 600840); c.2935G>A: p.(E979K) and c.2939A>T: p.(E980V) that co-segregated with ADNSHI in non-consanguineous families from Ghana and Pakistan. This study further confirms *SLC12A2* involvement in ADNSHI and that it should be included in targeted diagnostic gene panels. Our study emphasizes the urgent need of using exome sequencing to investigate HI in a wide range of populations including the understudied African populations, in order to improve our understanding of hearing pathobiology, locally and globally.

## Supplementary information

Supplementary materials
