# Supplementary material for: Further confirmation of the association of SLC12A2 with non-syndromic autosomal-dominant hearing impairment
Source: J Hum Genet. 2021 Jul 5;66(12):1169–75. doi: 10.1038/s10038-021-00954-6 (PMC8612923; doi:10.1038/s10038-021-00954-6)
Supplement: Supplementary file 1 — Supplementary materials [file 10038_2021_954_MOESM1_ESM.docx]

**Supplementary materials**

Table S1: Clinical significance prediction of the identified *SLC12A2* variants

| Bioinformatic Tools | c.2935G>A: p.(E979K) | | c.2939A>T: p.(E980V) | |
| --- | --- | --- | --- | --- |
|  | Score | Prediction | Score | Prediction |
| SIFT | 0.535 | Tolerated | 0.138 | Tolerated |
| Polyphen2 | 0.799 | Possibly damaging | 0.938 | Possibly damaging |
| MutationTaster | 1.000 | Deleterious | 1.000 | Deleterious |
| FATHMM | -1.900 | Deleterious | -2.02 | Deleterious |
| M-CAP | 0.063 | Deleterious | 0.074 | Deleterious |
| fathmm-MKL | 0.988 | Deleterious | 0.988 | Deleterious |
| MetaLR | 0.320 | Tolerated | 0.382 | Tolerated |
| PROVEAN | -0.140 | Neutral | -0.84 | Neutral |
| MutationAssessor | 0.805 | Low | 0.975 | Low |
| CADD score | 23.7 |  | 25.5 |  |
| ACGM classification for HI | PM2, PM1, PP1, PP3 | Likely pathogenic | PM1, PM2, PP1-S, PP3 | Like pathogenic |

Table S2: Excluded gene variants found in the two families

| Family | Gene | Variants | rs-number | Note |
| --- | --- | --- | --- | --- |
| GH-F4 | KCNQ4 | c.1125C>G: p.(S375S) | \| rs147168137 \| \| --- \| \|  \| | Synonymous variant No second variant found |
|  | OTOF | c.4377C>A p.(V692V) | rs374209343 | Synonymous variant No second variant found |
|  | BDP1 | c.2373C>T:p.S791S | rs200812578 | Synonymous variant No second variant found |
|  | SLC26A5 | c.1334T>C p.(I445T) | rs143428699 | Variant did not segregate |


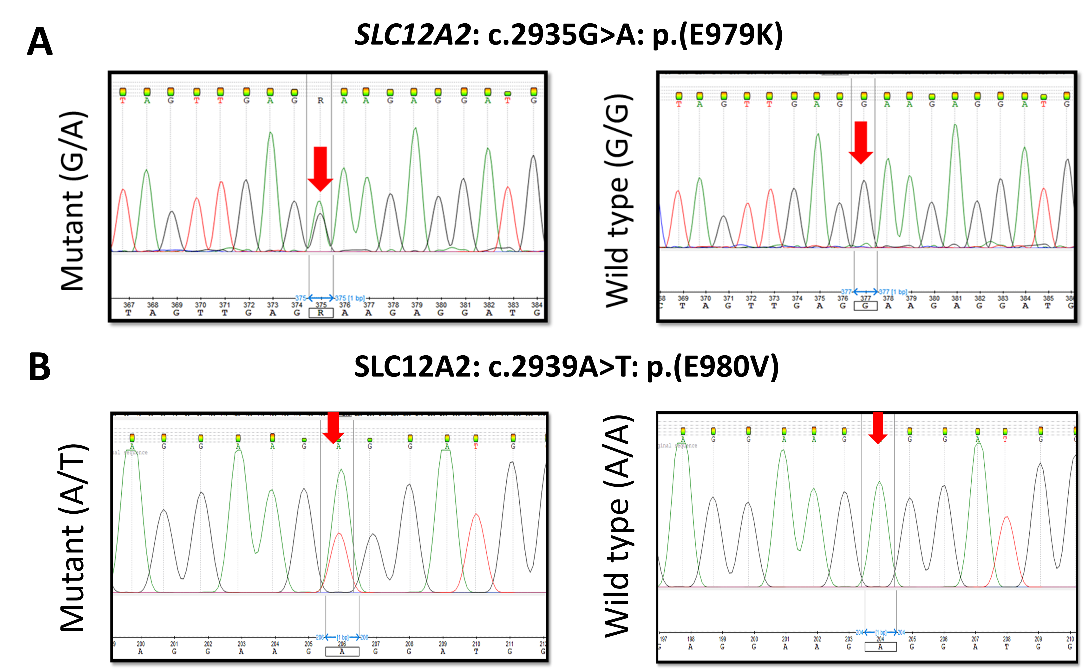


**Figure S1:** Sanger sequence confirmation of identified variants. Representative chromatogram of heterozygous (mutant) and homozygous (wild type) for (A) the *SLC12A2*: c.G2935A: p.(E979K) and (B) *SLC12A2*: c.2939A>T: p.(E980V).


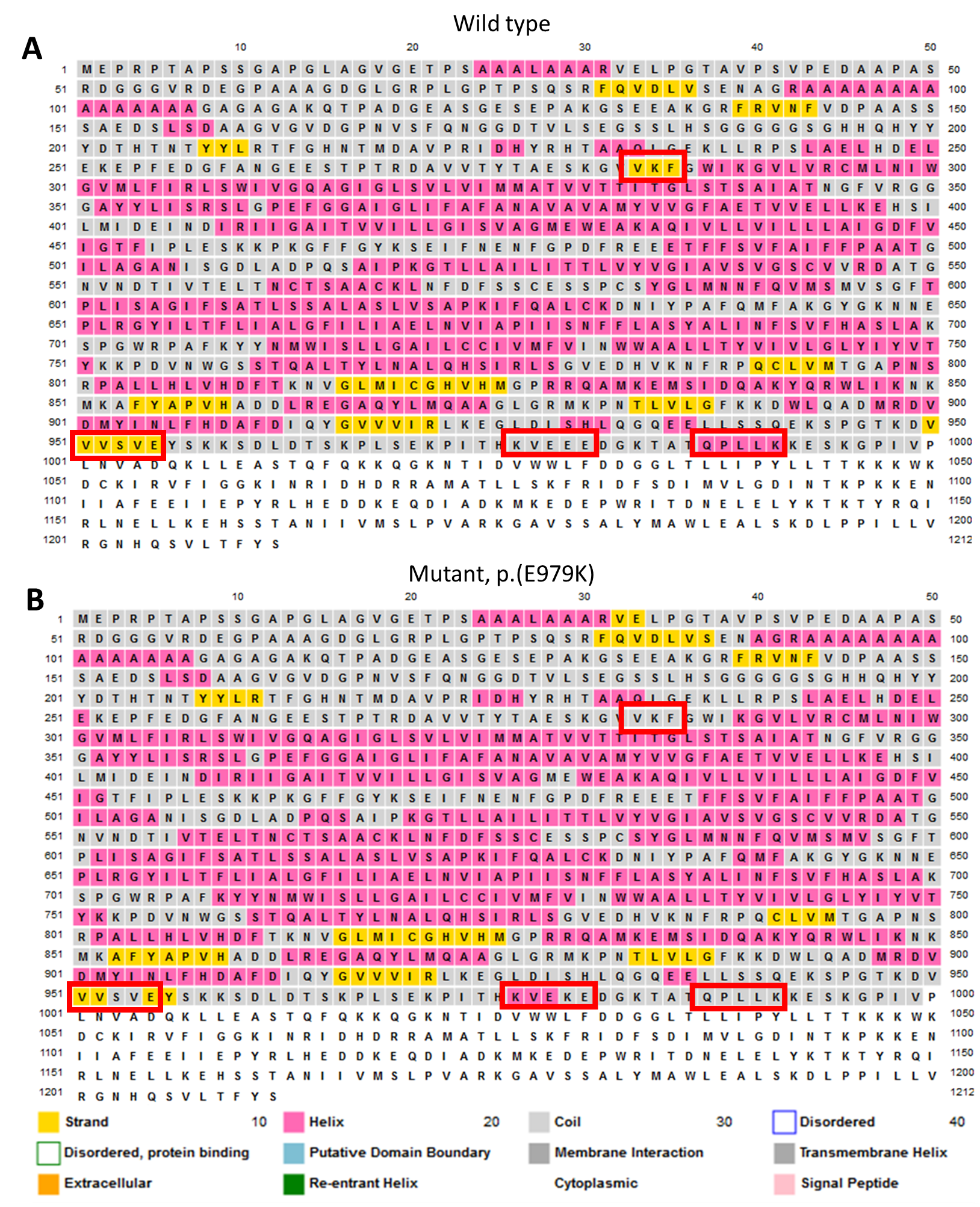


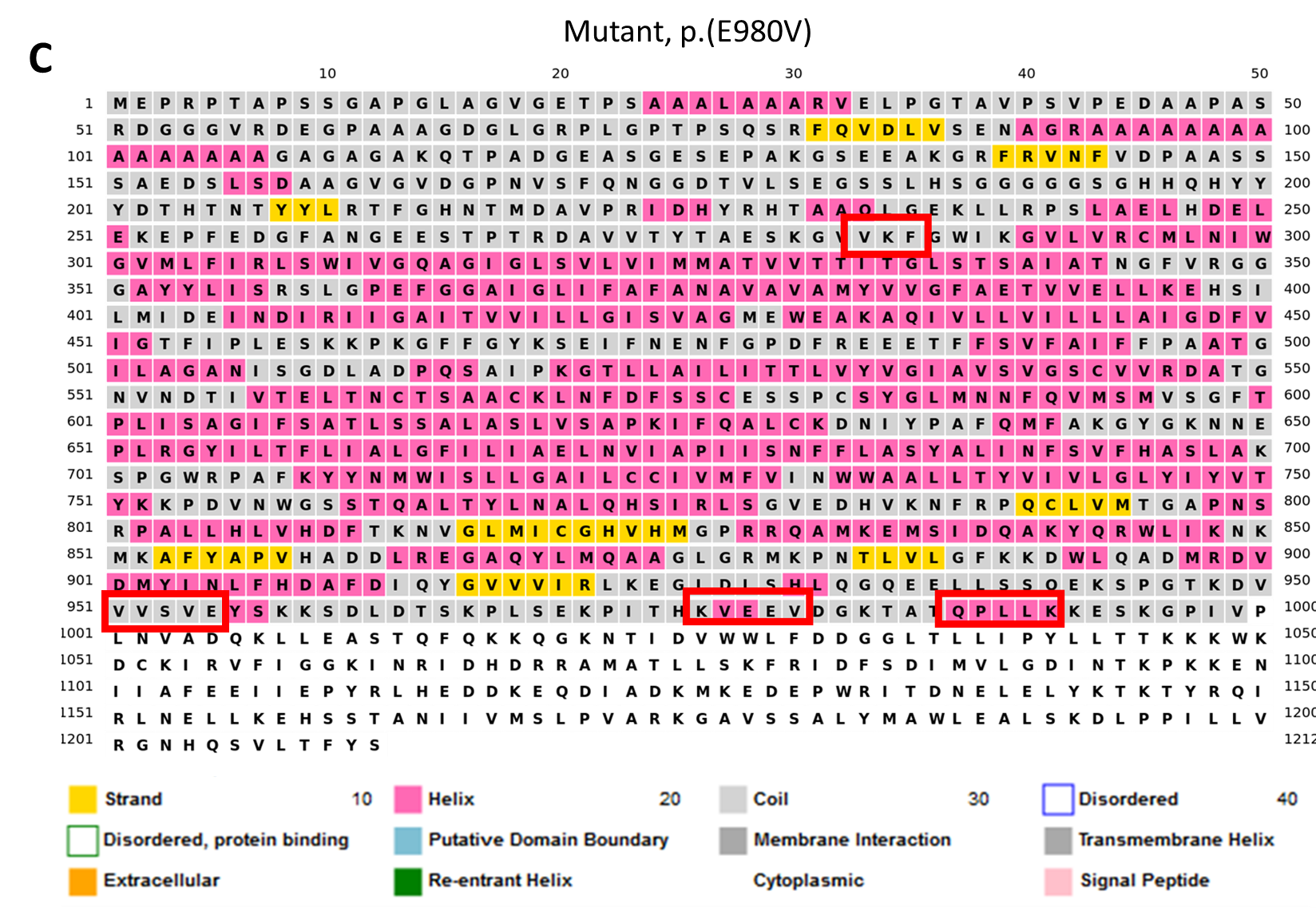


**Figure S2:** Secondary structure prediction using Psipred. The bioinformatic tool, Psipred, was used to predict the SLC12A2 secondary structures in the (A) wild type, (B) p.(E979K) mutant and (C) p.(E980V) proteins. Major differences were observed in the mutant compared to the wild type, and three of these were highlighted with a red rectangle.


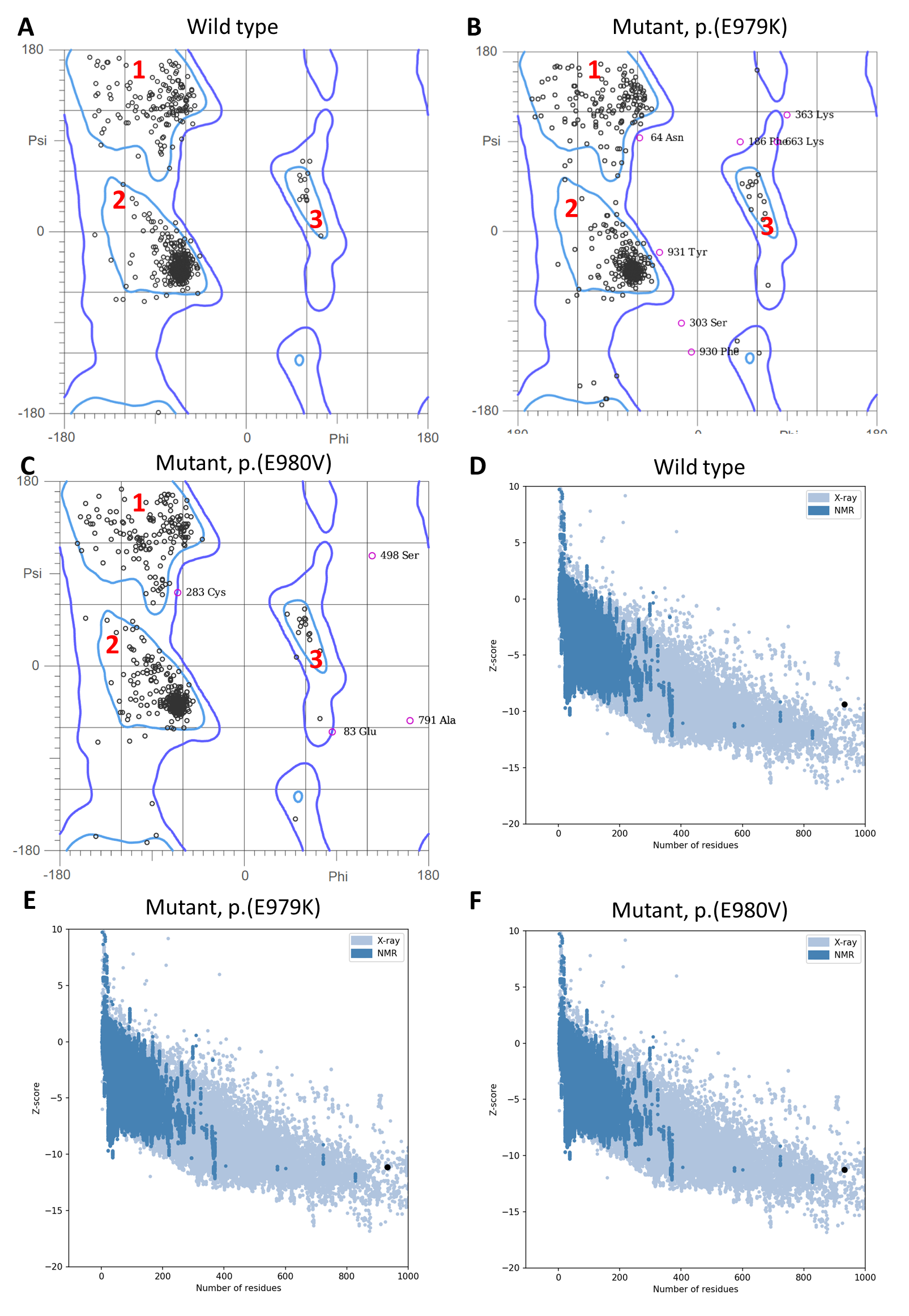


**Figure S3:** Protein model quality assessment. Ramachandra plots for (A) wild type, (B) mutant and (C) mutant p.(E980V) proteins. Plots of ProsA Z-score of the (D) wild type and (E) mutant p.(E979K) and (F) mutant p.(E980V) proteins. The areas marked 1, 2, and 3 on the Ramachandra plots are the allowed regions.
